# Using a Chatbot to Study Medication Overuse Among Patients Suffering From Headaches

**DOI:** 10.3389/fdgth.2022.801782

**Published:** 2022-03-17

**Authors:** Arthur Bézie, Valentin Morisseau, Romain Rolland, Arthur Guillemassé, Benoît Brouard, Benjamin Chaix

**Affiliations:** ^1^Wefight, Brain and Spine Institute, Paris, France; ^2^Hospital Gui de Chauliac, Montpellier, France; ^3^University of Montpellier, Montpellier, France

**Keywords:** chatbot agent, conversational agent, headache, overuse, medication

## Abstract

According to the World Health Organization, half the adult population around the world suffers from headaches. Even though this condition remains in most cases innocuous, it can have a major impact on the patient's quality of life but also on public health expenditure. Moreover, most patients manage their headaches on their own, without consulting a doctor. Therefore, self-medication can eventually lead to drug overuse, and consequently the emergence of a secondary disease called medication-overuse headache (MOH). The detection and follow-up of these unconventional patients represent a major challenge. Some of the latest technology advancements seem to be tailored and fitting for this context. The goal of this study is to investigate medication overuse in French patients suffering from headaches using the chatbot Vik Migraine. Data collection and analysis were assembled from answers to a questionnaire of 28 questions divided into three parts: socio-demographic profile, drug consumption, and medical follow-up. The study showed that medication overuse was often linked to increased headache frequency. Prescription drugs like triptans and opioids, were the most overused drugs among the cohort. This suggests that healthcare professionals could play a critical role in targeting these drugs in prevention of overuse.

## Introduction

### Medication-Overuse Headache

#### Context

Headaches are the most frequent neurological disorder in the world with an estimated prevalence of 50% among adults according to the World Health Organization (1). Even though this condition does not threaten patients' lives, it has a major impact in terms of quality of life. Headaches rank third in terms of Disability Adjusted Life Years and lead to an expenditure of 173 billion euros per year in Europe (2).

The third edition of the *International Classification of Headache Disorders (ICH)* has split headaches into three different groups: primary headaches, secondary headaches, and all other neuropathies, facial pains and other headaches.

The most frequent primary headache disorders are tension-type headaches and migraines while the most frequent secondary headache disorder is medication-overuse headaches (MOH).

According to the ICH, MOH are defined by three criteria (3):

- More than 15 days of headaches per month in a patient with a pre-existing headache disorder.
- Frequent overuse of acute and/or symptomatic headache drugs for more than 3 months (either 10 or 15 or more days/month, depending on the medication, whether it is acetaminophen, aspirin, other non-steroidal anti-inflammatory drugs, or any other acute or symptomatic headache drug).
- No other explanation of the secondary headaches.

Depending on the kind of drug involved in the overuse, we can distinguish eight different sub-forms of MOH. The possible drugs causing these headaches are ergotamine, triptans, analgesics such as acetaminophen and aspirin, opioids, combination analgesics (drugs containing an analgesic and another active substance), a mix of several of the previous drugs, unidentified drugs, or other drugs.

Some patients seem more at risk than others to develop MOH. The following are the risk factors that are being investigated (4–6) (Table 1).

**Table 1:** Risk factors for MOH and associated odds ratios identified in three studies [Hagen et al. (4), Scher et al. (5), and He et al. (6)].

**Risk factor**	**Odds ratio**
Age (under 50 y.o)	1.8
Gender (Female)	1.9
Low educational level	1.9
Chronic musculoskeletal complaints	1.9
Gastrointestinal complaints	1.6
Anxiety or depression	4.7
Smoking status	1.8
Physical inactivity	2.7
Metabolic syndrome	5.3
High daily caffeine intake	1.4
Tranquilizers' use	5.2
Aspirin's use	0.5
Ibuprofen's use	0.7
Opioids' use	2.3

#### Pathophysiology of Medication Overuse Headaches

Today, the exact pathophysiology for MOH is still unclear. However, some statistical tendencies have been identified. Somatosensory evoked potentials in the cerebral cortex of patients suffering from MOH show a hypersensitivity and responsiveness. Analgesic overuse is thought to create the same phenomenon through changes in the serotoninergic modulating system. Interestingly, this increased excitability of cortical neurons disappears after a drug withdrawal (4).

Moreover, functional imaging showed an altered connectivity in brain areas related to pain, cognition, addiction, awareness, and affective behavior. In patients suffering from MOH, gray matter volume was increased in some of these structures and lowered in the other areas. In fact, changes in gray matter volume have been shown to occur in many cortical and subcortical structures. Gray matter volume is increased in the PAG, bilateral thalamus, and ventral striatum, and decreased in frontal regions, including the orbitofrontal cortex, anterior cingulate cortex, the left and right insula, and the precuneus (7, 8). Because these areas are involved in nociceptive pathway, these observed abnormalities suggest an alteration in pain modulatory networks in patients with MOH. In addition, these modifications seem to be partially reversible after drug withdrawal.

**Figure 1:**
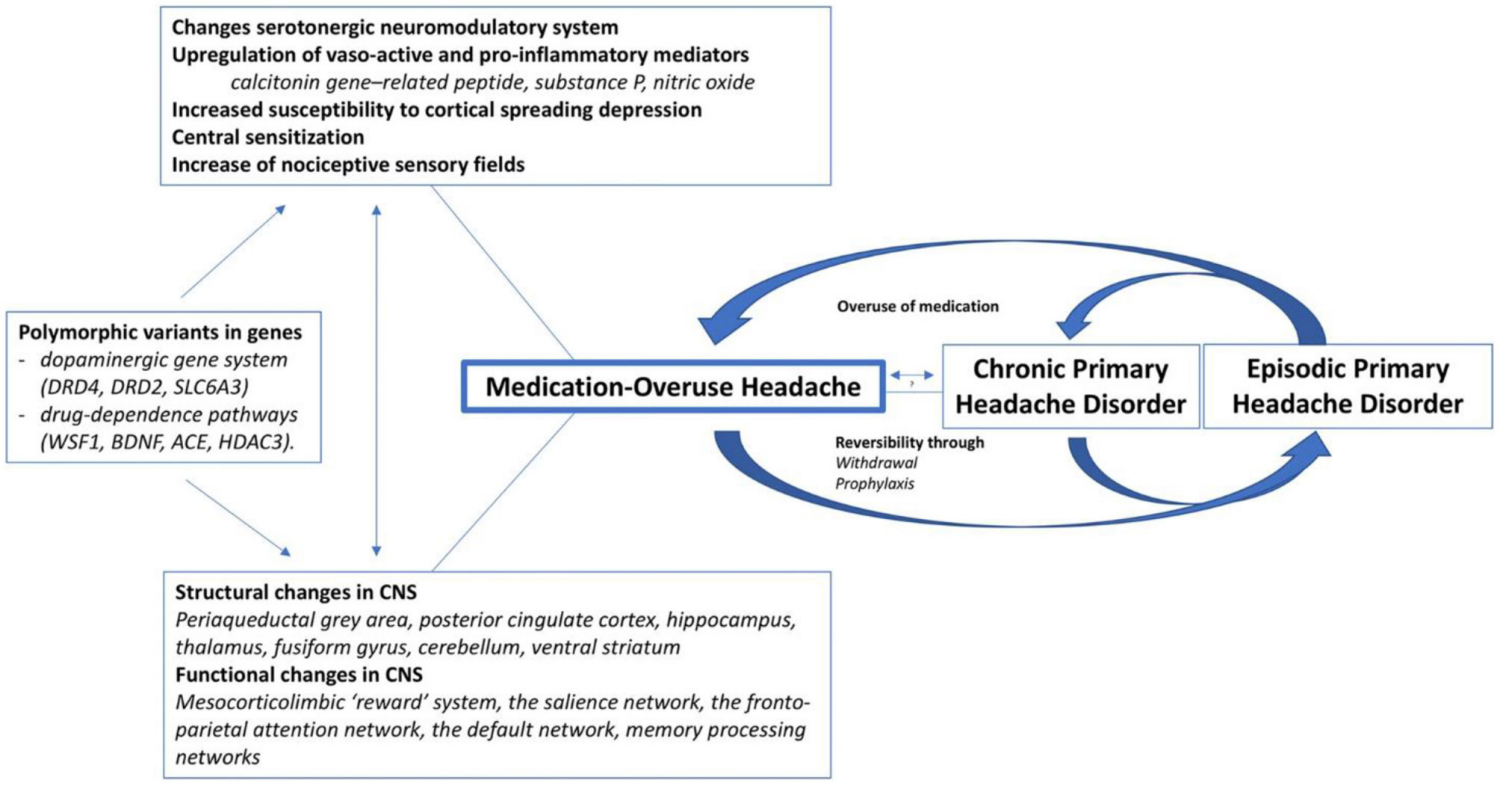
Current understanding of the pathophysiology of medication-overuse headache [Vandenbussche et al. (11)]. CNS, central nervous system.

Finally, some mutations (polymorphic variants) seem to be risk factors for developing MOH. In fact, considering the results obtained in recent studies (9, 10), genetic polymorphisms known to predispose abusive behaviors were found to be related to a higher drug consumption in MOH. This has also been highlighted by Cargnin et al. who described candidate polymorphic variants in genes of the dopaminergic gene system (DRD4, DRD2, SLC6A3), and genes related to drug-dependence pathways (WSF1, BDNF, ACE, HDAC3) (11) (Figure 1). The authors concluded that these traits are potential risk factors for MOH susceptibility or determinants of monthly drug consumption (12).

However, despite the performance of genetic studies in drug overuse and therefore MOH, high-quality evidence for genetic traits is currently lacking. Medication-overuse headache should consequently be considered as the progression of a specific subtype of migraine, containing within itself the start and cause of drug-overuse (13).

#### The Difficult Management of MOH

Medication-overuse headache remain an issue in France for several reasons. First, drug overuse is strongly linked to self-medication, especially in the case of over-the-counter drugs overuse and this issue is hard to monitor for healthcare providers. In fact, these patients are usually not followed by a general practitioner or a specialized doctor, and pharmacists cannot investigate the dispensing of every acetaminophen or aspirin. As a result, some patients are out of scope of the main factors contributing to a safe use of drugs.

In case of MOH, a drug withdrawal under medical supervision is recommended. The Brief Intervention for Medication-Overuse Headache (BIMOH) study found that brief intervention by the patient's own physician on the education of medication overuse had lasting and impactful results with 50% of headaches resolved within 3 months and 63% in 6 months (14). However, because of their pain, it may be hard for the patient to understand the finality of drug restriction on the short term. This explains the relapse rate of 40–50% in 6 years (15).

### Chatbots in Health

Chatbots are defined as digital tools that use machine learning and artificial intelligence methods to mimic human-like behaviors and provide a task-oriented framework with evolving dialogue able to participate in conversation (16).

This new technology can improve several aspects of a patients' health and comfort. For instance, a chatbot can answer questions at any time of the day, send reminders, avoid the feeling of discomfort for the patient when asking private questions. Additionally, it has been shown that chatbots can improve patients' compliance (17). Chatbots can also monitor patients and save time for healthcare teams for more complex tasks (18).

### Objectives

This study uses the chatbot Vik Migraine to study medication overuse among patients suffering from chronic headaches. It aims at identifying the therapeutic classes that are the most overused and at establishing a typology of patients according to their medication use.

## Materials and Methods

### Chatbot Design

Vik Migraine was designed by Wefight in 2018. It aims at helping patients suffering from headaches, or their relatives, by answering questions supported by quality-checked medical information. Users can also find content related to their condition and several features such as the treatment logbook. They can use Vik Migraine through Messenger or after downloading the Vik Migraine app, available on both iOS and Android.

Vik Migraine relies on both machine learning and natural language processing to understand the users' messages and render an appropriate answer.

**Figure 2:**
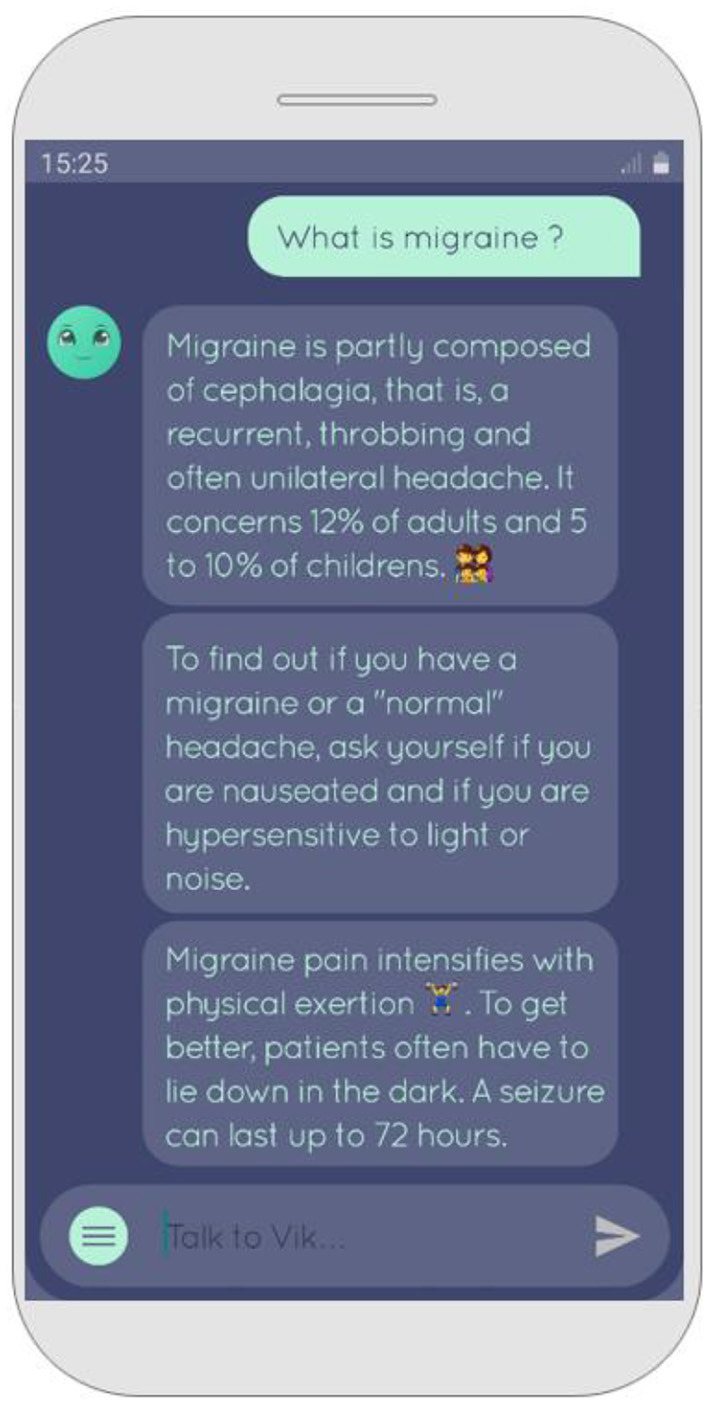
Chatbot's interface.

The chatbot interface is a conversation window. Patients and caregivers type their questions and the chatbot directly answers (Figure 2).

### Study Design

This is a non-interventional study. The questionnaire was sent to all patients using the Vik Migraine chatbot. They were filtered for eligibility based on the inclusion criteria (age >18 years, headaches). Participants were not paid. The data collected were anonymized and then hosted by Wefight on a server compliant with health care data storage requirements. Consent was collected online before the start of the study. In accordance with the French and European laws on information technology and civil liberties (Commission Nationale Informatique et Libertés, Règlement Général pour la Protection des Données), users had the right of use at their disposal to verify its accuracy and, if necessary, to correct, complete, and update it. They also had the right to object to their use and the right to delete these data. General conditions of use were displayed and explained very clearly, and they must be accepted before using the questionnaire.

### Outcomes

The impact of MOH on the patient's life was evaluated by the Headache Impact Test (HIT-6) score (20). It is based on answering a six-questions survey to rate the impact of headaches on the patient's life, and results vary from 36 to 78. The obtained score is sorted in four groups according to the mark: low impact (from 36 to 49), medium impact (from 50 to 55), high impact (from 56 to 59), and major impact (from 60 to 78).

This scoring range was chosen since it is capable of covering the impact of headaches and migraines, as well as providing unique and reliable information to the total score. In addition, for patients suffering from chronic migraine, the validity of the HIT-6 for measuring impact of headaches on daily life is supported (19). Moreover, the HIT-6 is an excellent tool for use in applied research, and clinical practice to assess patient experience, taking into account the short administration time, easy scoring, and interpretability.

### Statistical Analysis

Statistical analysis was performed using R, a programming language used for statistical computing. We also used FactomineR library to compute the multiple correspondence analysis (MCA).

The description of the included population was carried out by the calculation of average, standard deviation, median, and quartiles for quantitative variables, numbers, and percentages for qualitative variables.

A logistic regression was performed to identify the variables that are significantly linked to medication overuse. Odds ratios were based on this regression.

Multiple correspondence analysis was performed to cluster the population and determine the main parameters of each subgroup: five dimensions were used for clustering. The *v*-test values were computed with the FactomineR library and allowed a measure of association between variables. The *v*-test value is subject to a standard normal distribution so that a *p-*value can be calculated. The higher the value, more the modalities are correlated. In addition, the positive or negative nature of this value gives us information about its representation among the cluster (an over-representativity for positive values and an under-representativity for negative values). The shading of each used color represent a scale of representativeness: a low correlation (*v*-test between 0 and 5), a medium correlation (*v*-test between 5 and 10), and a high correlation (*v*-test greater than 10).

### Ethical Aspects

The protocol was approved by the Ethics Committee “Comité de Protection des Personnes *Ile-de-France X*.”

## Results

### Patients' Inclusion

**Figure 3:**
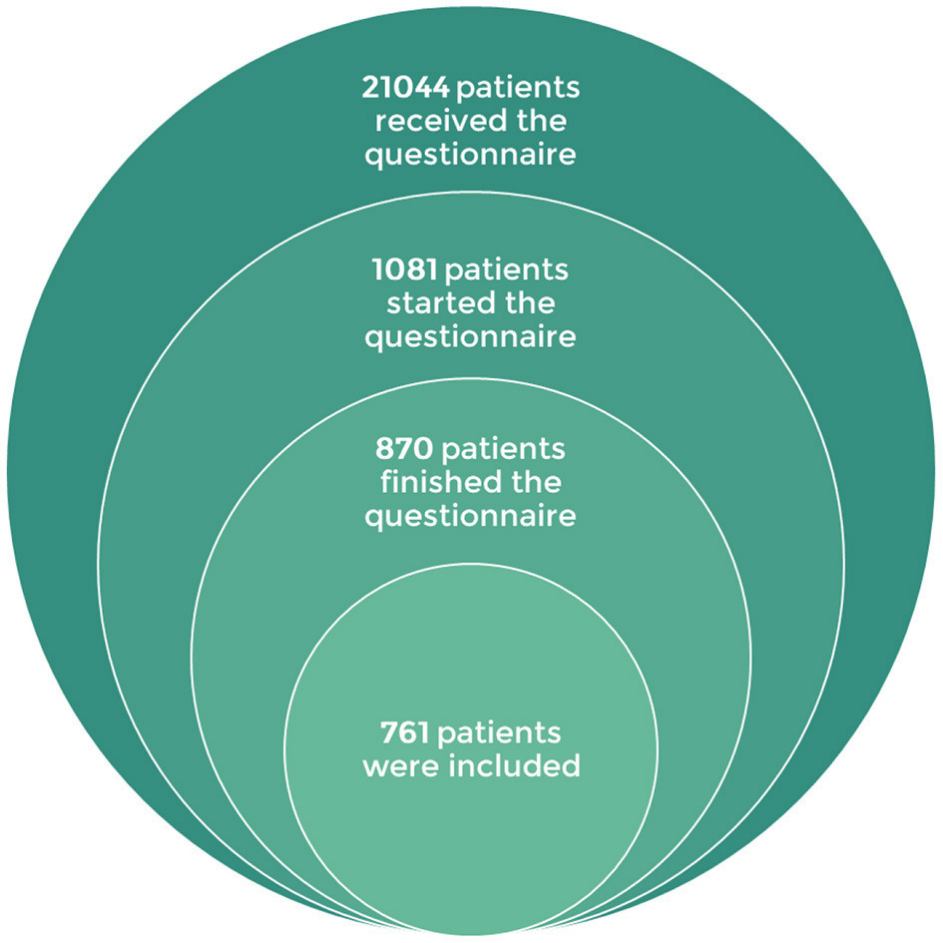
Stacked Venn diagram showing patients' inclusion.

The study was shared with over 21,000 users of Vik Migraine from 5 May 2021 to 28 May 2021. It was only sent to patients and not to caregivers. 5.13% (870) of the patients who received the questionnaire completed it. Among them, 109 were under 18 years old or not affected by chronic headaches and therefore were not included in the study (Figure 3).

### Vik Migraine's Patients' Profile

**Figure 4:**
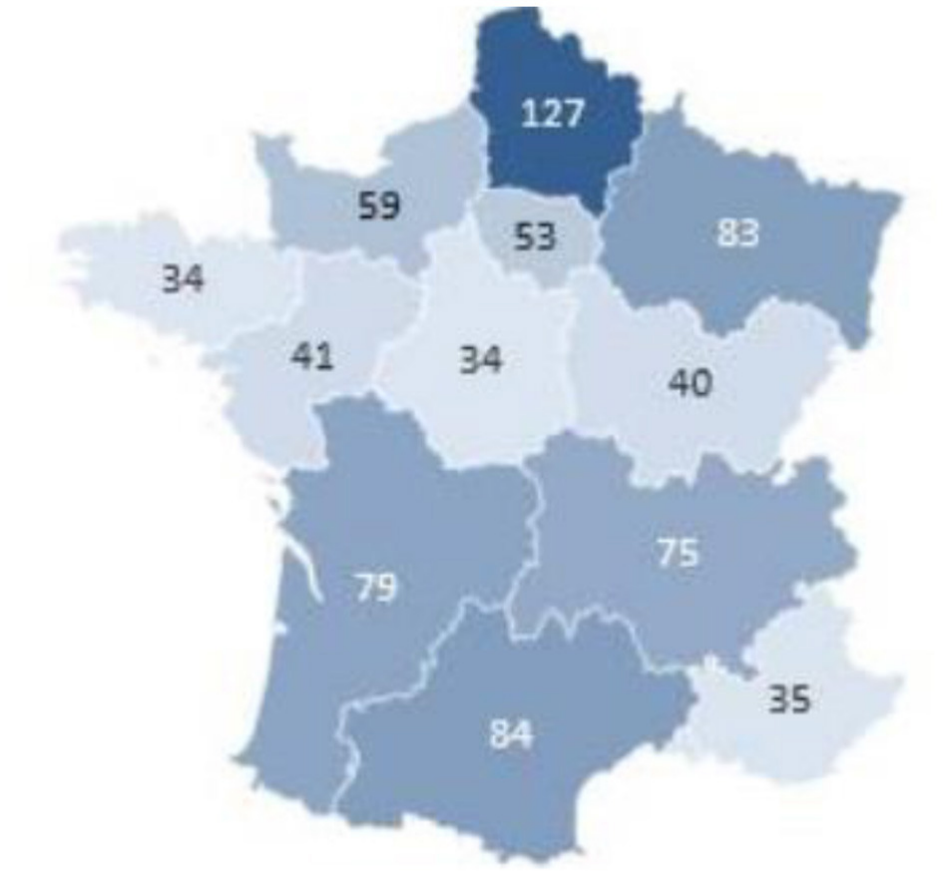
Geographical distribution of participants.

96.06% (731) of respondents are female. The cohort is young with an average age of 29.4 y.o. Patients included in the study were from 94 of the 95 French departments that constitute the country of France. The overall geographic repartition was heterogenous (Figure 4).

Most of the respondents are employed (38.2%, 291/761), unemployed (23.8%, 181/761), or students (21.7%, 165/761). The average number of comorbidities per patient is 1.29.

Forty-eight percent of headaches have been diagnosed as migraines while 41% are undiagnosed. Six hundred and eighty-eight respondents stated that they take acute treatments, namely painkillers or drugs to manage symptoms related to headache attacks. The average quantity of therapeutic classes is 1.68 per patient, mostly acetaminophen (58.9%, 405/688), NSAIDs (44.9%, 309/688), triptans (36.0%, 248/688), and opioids (35.6%, 245/688). Half of the respondents utilized alternative medicines such as homeopathy, aromatherapy, phytotherapy but also hypnosis, acupuncture, and cannabidiol.

### Binomial Logistic Regression

We studied 1 numeric variable and 22 qualitative variables in order to make a model able to assess the probability of drug overuse (Table 2). In fact, using the ICH definition, we gathered modality information of many entry variables to obtain the binary variable of interest regarding medication overuse. Thus, an intake of acetaminophen, aspirin or other non-steroidal anti-inflammatory drugs on more than 15 days per month; or any acute treatment for more than 10 days was considered as medication overuse.

**Table 2:** List of variables.

**Name of the variable**	**Class of the variable**	**Values**
Gender	Qualitative	Female, male, other
Age	Numeric	[18, 100]
Region	Qualitative	Any of the French regions
Socio-professional category	Qualitative	Company leader, employee, intermediate profession, manager, student, unemployed, other
Impact on everyday life	Qualitative	Low impact, mild impact, high impact, major impact
Comorbidities	Qualitative (multiple choice)	Neurological, endocrine, respiratory, joint disease, genetic, cardiovascular, infectious, other, no comorbidity
Smoking status	Qualitative	Non-smoker, former smoker, occasional smoker, habitual smoker
Drinking status	Qualitative	Non-drinker, several times a year, several times a month, several times a week, everyday
Exercise	Qualitative	No exercise, several times a month, several times a week, more than three times a week
Coffee	Qualitative	No coffee, sometimes, 1 cup a day, 2–3 cups a day, more than 3 cups a day
Diagnosis	Qualitative	Undiagnosed, migraine, tension-type headache, other
Headaches' frequency	Qualitative	>15 days/month, <15 days/month
Medication	Qualitative (multiple choice)	Acetaminophen, NSAIDs, triptans, opioids, ergotamine, metoclopramide, other, no medication
Self-medication	Qualitative	Self-medication, no self-medication
Number of intakes	Qualitative	1–2 intakes/day, 2–3 intakes/day, more than 5 intakes/day
Maximal number of days of medication intakes	Qualitative	1 day, 2 days in a row, 3 days or more in a row
Doctor in charge of the follow-up	Qualitative	No medical follow-up, GP, neurologist, other practitioner
Date of the last consultation	Qualitative	<6 months ago, 6–12 months ago, 1–2 years ago, more than 2 years ago
Alternative medicines	Qualitative	Alternative medicines, no alternative medicines

The model was improved by dropping terms one by one to maximize Akaike information criterion. Five qualitative variables were significant for the model: headaches' frequency (more or less than 15 days per month), triptans consumption (true or false), opioids consumption (true or false), number of drug intakes per day (2 or less, between 2 and 5, more than 5), and maximum number of days in a row with acute treatment consumption (1 day, 2 days, or more than 3 days). For each parameter, the odds ratio was calculated (Table 3).

**Table 3:** Odds ratios and confidence intervals of model's parameters.

**Parameter**	**Coefficient**	***P*-value**	**Odds ratio**	**Confidence interval [2.5%, 97.5%]**
Headaches' frequency >15 days/month	1.56	4.97 10^−15^	4.76	[3.23, 7.07]
Triptans = true	0.41	0.047	1.51	[1.00, 2.26]
Opioids = true	0.54	0.0078	1.72	[1.15, 2.56]
Number of intakes = 3–4 per day	0.32	0.128	1.38	[0.91, 2.09]
Number of intakes >4 per day	1.19	0.023	3.27	[1.18, 9.33]
Maximal number of days of medicine intake = 2 days	0.56	0.20	1.75	[0.78, 4.35]
Maximal number of days of medicine intake >2 days	1.64	5.92 10^−5^	5.14	[2.43, 12.26]

We notice that medication overuse is highly correlated with a high headaches' frequency. Taking acute treatments several times per day and during several days in a row are two behaviors often encountered in patients overusing drugs. The data results showed that triptans and opioids, which are prescription drugs, are the most overused.

### Multiple Correspondence Analysis

**Figure 5:**
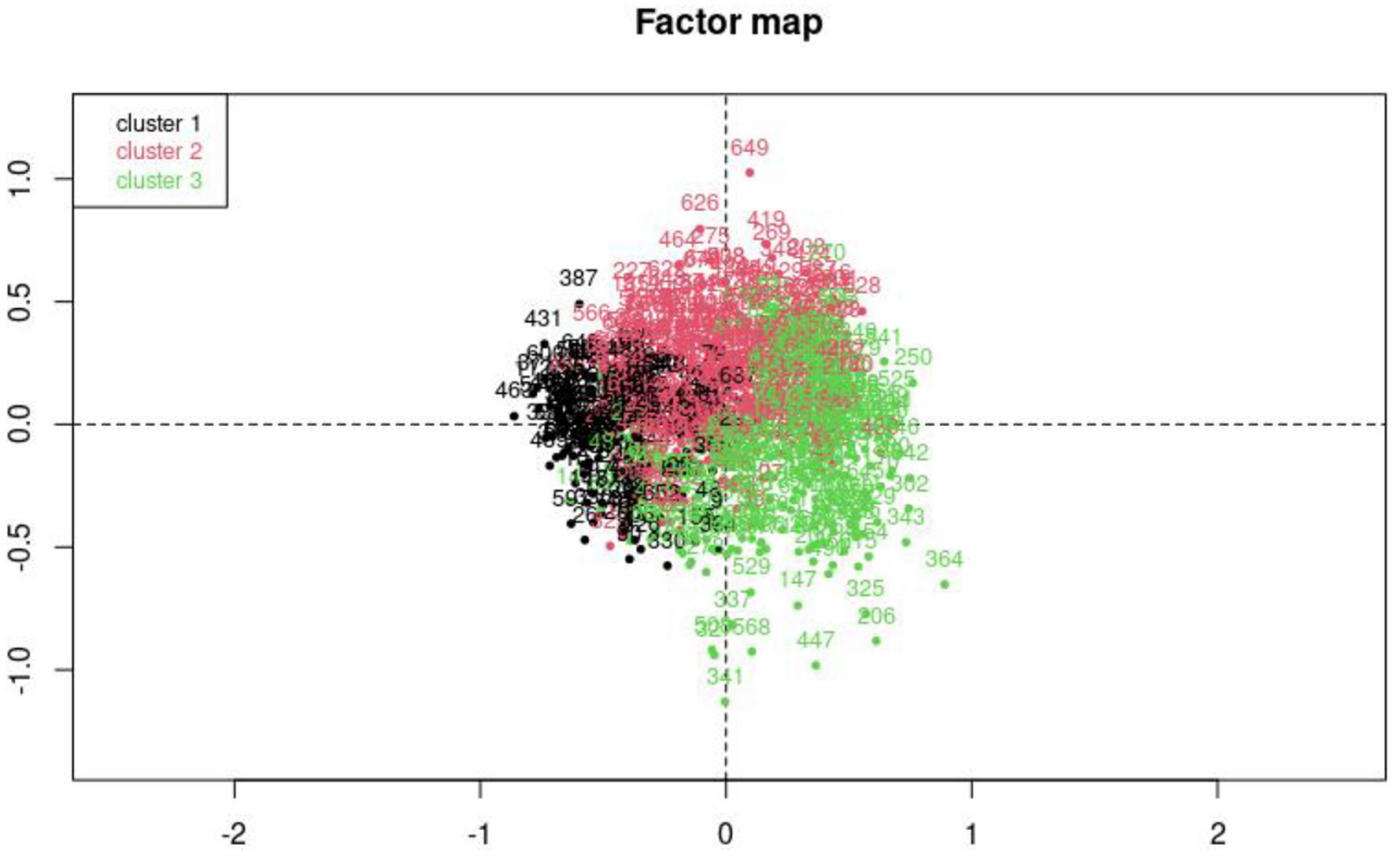
Factor map of the multiple correspondence analysis. Cluster 1, independent profile; Cluster 2, mixed profile; Cluster 3, medication overuse profile.

A MCA was performed to categorize the population in three clusters with different characteristics (Figure 5).

We also studied the importance of each variable for the clustering (Table 4).

**Table 4:** Variables ranked by order of importance for clustering.

**Variable**	***p*-value**
Doctor in charge of the follow-up	3.74e-43
Maximal number of days of medication intake	1.55e-37
Type of diagnosis	2.28e-36
Date of the last consultation	9.44e-35
Impact of headache on everyday life	2.68e-26
Medication overuse	2.91e-26
Headaches' frequency	3.56e-21
Socio-professional category	1.14e-16
NSAIDs' consumption	3.38e-15
Triptans' consumption	1.84e-14
Self-medication	4.91e-12
Neurological comorbidity	4.29e-11
Region	2.72e-10
Opioids' consumption	1.03e-9
Acetaminophen's consumption	1.93e-9
Regular exercise	2.51e-9
Alternative medicines	6.5e-8
Drinking status	1.39e-7
Gender	7.37e-7
Number of intakes	0.0000213
Respiratory comorbidity	0.0026
Coffee intake	0.00313

Medical variables were the most important for classification, whereas habits and profile variables seem to have little to no consequences on clustering.

Patients from cluster 1 are young (average age = 28 vs. 32 for cluster 2 and 3) and seem to manage their headaches on their own. Indeed, they are not followed by a practitioner and rely on self-medication, mostly acetaminophen. They suffer from headaches less than 15 days per month and do not overuse medication. The impact on everyday life is low, medium, or high. Besides, their use of acute treatments remains moderate, one day at a time with a few intakes per day. In this group, the proportion of undiagnosed patients is high.

Patients from cluster 2 also don't overuse medication and suffer from headaches less than 15 days per month. However, they are followed by a doctor and their last consultation is often between 1 year and 6 months ago. Most of them have a job and their main acute treatments are NSAIDs. This group is also correlated with self-medication and alternative medicines. The trends in this cluster are less strong than in other clusters and the absolute value of *v*-test statistics never exceed 7.96.

While patients that don't overuse drugs are shared between cluster 1 and 2, cluster 3 contains most of the patients with medication overuse (Table 5). The latter is associated with frequent headaches and several days of drug intakes in a row. They tend to use opioids and triptans. These patients are followed by a GP or a neurologist and their last consultation to discuss their headaches took place within the last 6 months. The impact on everyday life is severe, they are more likely to suffer from MOH and to have neurological comorbidities than respondents within clusters 1 and 2.

**Table 5:** *V*-test's statistic for main variables and for each cluster.

**Parameter**	**Cluster 1**	**Cluster 2**	**Cluster 3**
Medication overuse = *True*	−6.69	−5.65	10.6
Alternative medicines = *True*	−3.13	5.68	−3.2
Diagnosis = *Migraine*	−10.3	2.69	5.57
Diagnosis = *Tension-type headache*	−4.61	3.84	−0.764
Diagnosis = *Undiagnosed*	13	−4.42	−6.41
Medication = *Acetaminophen*	5.88	0.0529	−4.8
Doctor in charge of the follow-up = *GP*	−9.97	2.76	05.01
Doctor in charge of the follow-up = *Neurologist*	−5.77	−2.92	6.72
Doctor in charge of the follow-up = *No medical follow-up*	13.2	−1.98	−8.83
Headaches' frequency = *>15 days per month*	−5.03	−5.61	9.61
Impact on everyday life = *Low impact*	2.41	0.318	−2.83
Impact on everyday life = *Major impact*	−10.1	3.42	6.02
Impact on everyday life = *Mild impact*	7.2	−3.71	−3.4
Impact on everyday life = *High impact*	5.69	−1.57	−3.74
Date of the last consultation = *>2 years ago*	10.2	−3.52	−5.65
Date of the last consultation = * <6 months ago*	−6.43	−3.27	8.59
Date of the last consultation = *6–12 months ago*	−3.85	6.48	−3.67
Maximal number of days of medication intakes = *1 day*	9.62	−3.85	−5.42
Maximal number of days of medication intakes = *2 days in a row*	1.87	4.1	−5.83
Maximal number of days of medication intakes = *3 days or more in a row*	−9.35	−1.37	9.1
Comorbidities = *Neurological*	−3.01	−4.68	6.79
Medication = *NSAIDs*	−1.68	7.96	−6.68
Number of intakes = *1–2 intakes/day*	4.91	−2.93	−0.999
Number of intakes = *3–4 intakes/day*	−4.11	2.92	0.371
Number of intakes = *>5 intakes/day*	−2.7	0.205	1.73
Medication = *Opioids*	−5.95	−0.298	5.01
Comorbidities = *Respiratory*	−2.9	−0.7	2.96
Self-medication = *True*	−4.09	7.33	−4.31
Socio-professional category = *Intermediate profession*	−2.73	4.18	−2.27
Socio-professional category = *Manager*	−2.87	6.53	−4.79
Socio-professional category = *Student*	1.15	3.27	−4.4
Socio-professional category = *Unemployed*	1.14	−6.14	5.02
Medication = *Triptans*	−8.11	0.86	5.37

*The colour level shows density*.

## Discussion

This article highlights and emphasizes the suitability of conversational health chatbots, as a concrete and relevant tool for conducting real-world-data-based descriptive studies with ease and great patient responsiveness. In fact, the questionnaire was live on the Vik migraine app for a period of 2 weeks and gathered engagement from 870 patients who completed the questionnaire. This allowed the studies and data teams to collect a large amount of data in a short period of time. However, some selection bias must be considered, such as the fact that a majority of the patients were young women.

Both logistic regression and MCA stress the fact that opioids are often involved in medication overuse. This was shown by Sher et al. (5) in the United States and the associated odds ratio was similar to the one calculated here. However, the level of association between triptans and medication overuse had not been measured until now.

The ICHD definition of medication overuse considers the number of days of medication consumption per month, patients overusing drugs were therefore expected to take drugs for several days in a row and the quantity of medication intakes per day is not directly linked to the definition of drug overuse. However, it is evident through our results that some patients overusing medication not only take drugs many days in a row, but also consume a higher quantity of drugs than other patients within a day. Therefore, the severity of the issue we are addressing in this study is highlighted by the excessive drug consumption and the participation of both excessive daily and monthly intakes of drugs in the process of overuse.

Overconsumption of drugs appears to be associated with a high frequency of headaches' attacks. It is also considered one of the causes of frequency augmentation. According to the ICHD, if the use of medication increases the frequency of attacks while no logical explanation exists, the patient suffers from MOH. Almost all patients from cluster 3 may suffer from MOH without knowing it.

Therefore, drug abuse is a key element in the cause-and-effect cycle, as a two-way connection, linking painful and frequent primary headaches to MOH. Concerning its methodology, our descriptive study is not able to distinguish if the interviewed patients are overusing drugs because of their headaches or if they are suffering from headaches because of their medication overuse. Taking this into account, it would be interesting to conduct follow-up studies regarding this matter by comparing the severity and frequency of headaches before and after drug withdrawal, notably by involving neurologists who would diagnose MOH among patients and make sure there is no other underlying cause for their headaches.

Nevertheless, challenging some preconceived ideas, the data results showed that frequently consulting the doctor and avoiding self-medication do not seem to protect from medication overuse. In fact, people from cluster 1 dealing with their headaches on their own do not overuse drugs while patients from cluster 3 that frequently see their general practitioner tend to take too many medicines. Some hypotheses can explain this: First, people from cluster 3 may suffer from intense headaches that need to be alleviated by opioids that are known to trigger addiction. Second, Cluster 3 individuals may have an increased sensitivity to pain perception because of a derangement in their central pain modulating system, resulting in them taking more medication to ease their pain. Finally, they may need a different preventive treatment or a more supportive lifestyle to manage and therefore diminish the intensity and frequency of their headaches which may cause them to overconsume acute medication.

## Conclusion

This descriptive study executed through the Vik Migraine application allowed us to identify the profile of patients suffering from primary headaches. We were, in turn, able to detect the rate of secondary headaches caused by medication-overuse and therefore the profile of patients suffering from MOH. The results showed that medication overuse was often linked to an increase in headache frequency and that certain prescription drugs, especially triptans and opioids, were the most overused drugs among the cohort. This highlights the role that healthcare professionals could play in targeting these drugs in prevention of medication overuse and its outcome. This study has also exposed the need for a clear outline concerning specific treatment plans for patients at risk of developing MOH and raising patients' awareness on these types of headaches.

## Data Availability Statement

The original contributions presented in the study are included in the article/supplementary files, further inquiries can be directed to the corresponding author/s.

## Ethics Statement

The studies involving human participants were reviewed and approved by Comit de Protection des Personnes Ile-de-France X. The patients/participants provided their written informed consent to participate in this study.

## Author Contributions

AB and VM: study concept and acquisition of data. AB, RR, and BC: drafting of the manuscript and supervision. AG: statistical analysis. AB, VM, and AG: interpretation of data. All authors: critical revision of the manuscript for important intellectual content.

## Funding

This research was supported by Wefight.

## Conflict of Interest

AB, VM, RR, AG, and BB are employees of Wefight. BC own shares of Wefight.

## Publisher's Note

All claims expressed in this article are solely those of the authors and do not necessarily represent those of their affiliated organizations, or those of the publisher, the editors and the reviewers. Any product that may be evaluated in this article, or claim that may be made by its manufacturer, is not guaranteed or endorsed by the publisher.
